# The Antrum of Highmore and Its Diseases

**Published:** 1872-03

**Authors:** Leon. F. Harvey


### ARTICLE VI.

### *The Antrum of Highmore and its Diseases.*

BY LEON. F. HARVEY, M. D., D. D. S.

[Read before the Eighth District Dental Society.]

Perhaps an examination of the history and anatomy of the antrum of Highmore, will be interesting as a preliminary to the consideration of its diseases. We find that its name was given to it by Nathaniel Highmore, a physician and anatomist, who was born at Fordingbridge, in Hampshire, in 1613, and educated at Trinity College, Oxford. He took his degree of M. D. in 1642, and settled at Sherburn, in Dorsetshire, where he became eminent in the practice of the profession. Highmore was a theorist from necessity, the opportunities for practice in those days being few and far between. In publishing the description of the Antrum Highmorianum, he led to the discovery of a disease until then unknown, and to an operation, which was not attempted, however, until fifty years after. He also noticed that the alveolars penetrated into the cavity, and and that they were only separated by a thin scale. High more published *Corporis Humanis Disquisitio Anatomica* fol. 1651 ; *The History of Generation*, 8 vo., 1651 ; *De Hypocondriaca Affectione*, 12 mo., 1660. He died in 1685. It is termed antrum as similar cavities are whose entrance is smaller than the bottom of the cavity. Its situation is in the superior maxillary bone above the bicuspid and molar teeth, the fangs of which rise above the floor, or perhaps we should say form its floor, they being covered by bone. It has an opening into the middle meatus of the nose, the form of its cavity being conical. Its lining membrane is a continuation of the mucous membrane of the nose, known as the Schneiderian. Conrad Schneider, who had the honor of attaching his name to it, was an anatomist of Wittemburg. In 1661, being a contemporary of Highmore, he published a treatise entitle *De Catarrhis.* Up to his time it was supposed that the secretion of the nose came from the brain, and he announced to the world that it emanated from the membrane of the nose. This membrane is also known as the Pituitary. There is a constant secretion taking place in this cavity which escapes through the middle meatus into the nose. The arteries of the antrum are the superior dental, and the infra orbital, both being branches of the internal maxillary artery, one of the terminal branches of the external carotid. The anterior, middle and posterior dental nerves may be found in the antrum, running under the mucous membrane, giving off branches at different points.

This cavity is liable to several diseases; the first which we will consider, is the result of inflammation from some cause, known or unknown—a purulent discharge from a dead tooth, a blow upon the face, an extension of a severe catarrhal attack may all produce inflammation at this point. It may exist for years and yet give the patient but little nconvenience; at last the accumulation of muco-purulent matter may be so great that pressure on the walls takes place and a slight protuberance is observed, and if exit is not given to the fluid, nature forces an opening through the bone and integument. At first there is a dull, heavy pain, the feeling being described as if everything was filled up and a heavy pressure was constantly kept up. When the inflammation is at its height, there are throbbing and lancinating pains. If there is not an escape of muco-purulent matter by way of the nose, an opening should be made through the floor of the antrum by extracting a bicuspid or molar tooth; and should that not lay it open, a drill should be used to finish the operation. Of course in the extraction of the tooth, its removal will depend on its condition; taking that one, the loss of which will be least felt, or possibly its loss prove a gain. Should a dead tooth be the cause of the trouble, that one should certainly be removed. In this disease it is frequently the case that the opening into the meatus is entirely closed ; if such be the condition the artificial opening should never be allowed to close till communication is perfectly established between the antrum and the nostrils. If the pressure be very great, the lachrymal duct may be obstructed and the fluids of the eye will flow down the cheek. The discharge oftentimes is very offensive. An entrance being made, the cavity should be thoroughly cleansed out when the discharge has ceased, by gently injecting warm soap suds made with castile soap, after which, use warm water in which a few drops of Tinct. Calendula have been dropped. This having been done, a weak solution of Iodine or Chloride of Zinc should be thrown into the antrum. The lees of wine or port wine may also be used to advantage. This should be repeated every day, or every other day after a few weeks, till all discharge ceases, the purpose being to remove all inflammation and establish a healthy secretion. The antrum should be syringed out every time an application is made. If the general health is impaired, tonics must be prescribed ; and should there be a scrofulous diathesis present, it would be well to use the course of treatment generally observed in such cases. There should be left in the opening a pledget of cotton to keep it open.

Abscess of the antrum is sometimes observed, though very rarely. If there be pus present, which must always be the contents of an abscess, we shall have the characteristic signs due to its presence ; rigors and lancinating pains. Sometimes there will be fluctuation of the part. The same course must be pursued here, namely, to make an exit for the matter. Necrosis may take place in the case of an abscess. If this occur, the bone should never be removed till complete separation has taken place. If the swellIng of the face exist after a removal of the disease, it may be reduced or entirely removed with a roller and bandage.

Some of the cases yield readily to treatment, especially recent ones, but many will be found to demand a long course of medication.

Mrs. H., æt. 45, came to us after having been treated for some time for catarrh, having suffered from a difficulty in the region of the antrum for four or five years. On examination there was observed some swelling on the right cheek; the patient had been annoyed for a long time with a profuse discharge, very offensive, from the nose, muco-purulent in character—the pain at times was very severe, so much so as to keep the patient awake nights, extending even to the lower jaw. When we saw her, the discharge through the nose had ceased. Feeling confident that an opening made into the antrum would in a measure relieve the pain, the first molar was extracted, from which opening there was a copious discharge of muco-purulent matter, much to the relief of the patient.

We then injected into the cavity warm water and castile soap, after which warm water impregnated with creosote, the cavity being washed thoroughly every day; astringents were used, as Tinct. Myrrh, Chloride of Zinc, &c.

Continuing this treatment for six months the trouble was entirely removed. The opening was kept open with pledgets of cotton, which very frequently gave much trouble by getting into the antrum.

I am indebted to Dr. Watt, of Cincinnati, for the following suggestion in regard to the use of a tent. Taking a small piece of slippery elm bark, he cuts it to fit the opening made in the antrum, and to it attaches a ligature. With this tent he closes the antrum and allows the ligature to lie on the gum. Should the tent get into the antrum, it can be readily removed by the use of its silk attachment.

Mrs. A., æt. 35, said she had been troubled for two or three months with a slight pain at times in the upper jaw, there was a constant discharge of an offensive odor from the nostrils, no swelling. Extracted the second bicuspid and there was immediate relief with the copious discharge.

Followed about the same treatment as in the last case, discharging the patient, however, in a much shorter time.

Mrs. S., æt. 30, fell, striking on her face. Soon after, she had every symptom of disease being present in the antrum, even to an offensive discharge. With all the facts before us, it seemed certain that the difficulty lay in the antrum, and therefore the second bicuspid was extracted, but no discharge followed and there was no relief from the continued pain. All the usual remedies were made use of but they were of no avail. Lately we have heard from the patient, who now resides in another State, and have learned that she is still a great sufferer, and it is now supposed that the trouble all arose from the bite of an insect. This last case we refer to, to show the liability to error in the diagnosis of affections of this cavity.

Tumors are very often found growing in the antrum, and their diagnosis is at times difficult. They may be fibrous, osseous, fatty, cartilaginous, erectile, or encephaloid. The former is of the most frequent occurrence, and is most frequent in patients of advanced age. Next to these come the encephaloid. The osseous are remarkable for their attempts to eliminate themselves by necrosis. Tumors of all kinds find their origin in the mucous membrane of the antrum. They will produce a protuberance according to their density. Some attain to a very large size. As the size increases, the nasal cavity is very frequently obstructed, deglutition and respiration are impeded, and an upward pressure towards the eye produces disorders of that organ. How are we to distinguish a tumor from fluid accumulations? We are to ascertain if there are any projections beyond the walls of the cavity. If it be a tumor, after a time it will present itself to the surface. As a rule it will insinuate itself between the bones, pressing them apart, if it be encephaloid in character. Sometimes the adjacent glands may be affected by a malignant tumor. It is observed that simple tumors occur more frequently in the young, whilst the malignant forms are to be found in middle or advanced life.

I shall cite one case here to show the difficulty of the diagnosis. It was under the care of Mr. Ferguson of Kings College Hospital, Marianne S., æt 25, was admitted in the Adelaide ward March, 23, '50; was born in Hampshire and had always lived in the country. Her health had never been strong; she had always been looked upon as delicate, and was subject to headaches. About eight months before, a swelling appeared on the right cheek in the situation of the antrum about three-quarters of an inch external to the right ala nasi, and on a level with that process. It came on imperceptibly without any pain and when she first noticed the tumor it was about the size of a pea, immovable and not painful on pressure. Since that time it has gradually increased, but of late somewhat rapidly. On admission, the tumor was about the size of a hen's egg, involving the whole of the non-alveolar portion of the superior maxilla on the right side. Externally it formed a large smooth undiscolored projection, drawing the eye a little downwards. On passing the finger inside the cheek and along the outside of the teeth, the quantity of the gum between the latter and the projection of the swelling was felt to be very small. On looking into the mouth the right half of the hard palate presented instead of the normal cavity, a smooth convexity rather more than an inch long and half that width, this convexity lying between the median line and the gum of the molars and bicuspids of that side. On pressure, which gives the patient no pain, there is a feeling of some fluctuation and the hand experiences an elastic tense sensation. When strong pressure is made on the cheek the patient feels the thrust downwards. The first and third molar and the first bicuspid are destroyed by caries and the other teeth are likewise affected; in fact the patient has always suffered from toothache and she has hardly a sound tooth in her head. The zygomatic muscles have been so stretched that they have become paralyzed, and on smiling, the right corner of the mouth instead of being drawn upwards is pulled downwards and outwards, the triangularis oris muscle alone acting. The most yielding part of the tumor is the situation where it first appeared. Five days after admission the patient was attacked with severe inflammation on the left side of the face, involving the glands and gums. It was sometime before she recovered from this complication, the disease of the antrum remained, however, in the same state as before described. Mr. Ferguson, considering that the time had now arrived to adopt operative measures for this affection, had the patient brought into the theatre on the 27th of April, and determined to proceed to the removal of the superior maxilla, if the cavity of the antrum were really, as suspected, found occupied by a solid tumor. Bearing, however, in mind that the appearances presented might be owing to an accumulation of fluid, the operator resolved to make an exploring puncture through the gum before any further steps were adopted. An exploring needle was therefore passed into the tumor, and when fluid had been seen to escape, Mr. Fergusson took up the scalpel and made an incision through the gum down to the anterior wall of the antrum and took out a portion of the same, which was very much thinned. By this proceeding a quantity of glairy fluid, containing flakes of cholesterin was let out of the antrum, and the nature of the tumor being thus ascertained, no further interference was required, and the patient removed. The swelling went on diminishing in size for the next few weeks, and the patient was discharged on the third of May in a pretty satisfactory condition.

Here was a good surgeon ready to make an operation for a tumor when there was really only fluid present. Gensoul, a noted surgeon, has been known to make incisions in the cheek when only an antral abscess was present. In the diagnosis, the puncture should be made to be sure with what you have to deal. The only treatment for tumors of the antrum is extirpation. This the dentist, unless he makes a specialty of removing tumors, will not do, and the case is sent to a surgeon. Be careful that you do not send a diseased antrum or an antral abscess to be operated upon for a supposed existing tumor. There may be a collapse of the antrum from disease occurring within it. For this there can be no remedy as in the expansion. In some cases the deformity is quite marked.—*Dental Advertiser.*

---

